# The Medical School of Philadelphia

**Published:** 1852-11

**Authors:** 


### THE MEDICAL EXAMINER.

PHILADELPHIA, NOVEMBER, 1852.

### THE MEDICAL SCHOOL OF PHILADELPHIA.

It gives us great pleasure to state that an unusually large class of students is in attendance at the various institutions, which compose the great medical school of Philadelphia. The benches of the University, and of the Jefferson College, are both crowded to overflowing, and a very marked and decided increase is noticeable in the numbers at the Pennsylvania Medical College.

In connection with the University, the point of chief interest during the present session has been the début of the new Professor of Chemistry, Dr. Robert E. Rogers, who fills the chair vacated by the death of his lamented brother, Dr. James B. Rogers. Dr. Rogers has proved a most impressive lecturer and effective teacher, and has well maintained the high reputation which accompanied him from the University of Virginia. His course elicits unqualified satisfaction from the class, and his appointment cannot fail notably to promote the interests of our Alma Mater.

The present session at the Jefferson College bids fair, we believe, at least to equal in success the brilliant career of the last and previous years. Nothing occurs to vary the unbroken unity which the able and judicious faculty have so long maintained.

That the Pennsylvania College must evince a striking impetus from the infusion of strength in its reorganization last summer, we confidently predicted; and our anticipations have been fully realized, both in the distinguished success which has attended the efforts of the new professors, and in the large accession of students to the institution.

The clinical and various private courses are, we believe, unusually well attended; and in all the minor branches of the great business of medical instruction an encouraging success is witnessable. We may say, with the most entire truth, that Philadelphia this year fully maintains her ancient supremacy as the metropolis of American medicine. B.

---

